# Supplementary material for: Alterations in RNA Expression Profile Following S. aureus and S. epidermidis Inoculation into Platelet Concentrates
Source: Int J Mol Sci. 2025 Mar 26;26(7):3009. doi: 10.3390/ijms26073009 (PMC11988392; doi:10.3390/ijms26073009)

Supplementary Figure S1. Principal component analysis (PCA) plots of gene expression profiles of platelet concentrate after *S. aureus* inoculation. (A) 0-hour and 1-hour after *S. aureus* inoculation. (B) 0-hour and 3-hour after *S. aureus* inoculation. (C) 0-hour and 6-hour after *S. aureus* inoculation.

A

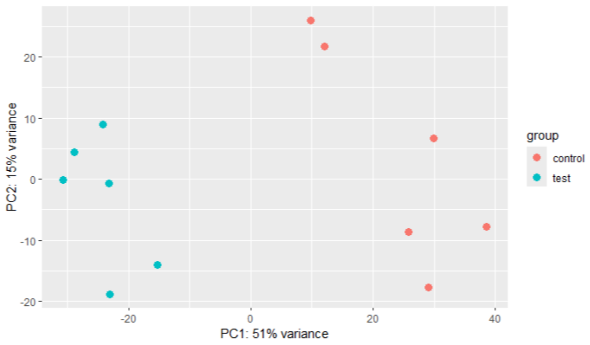

B

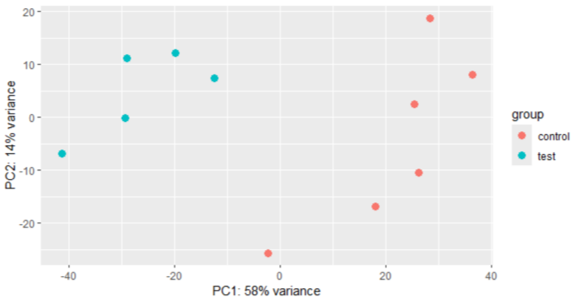

C

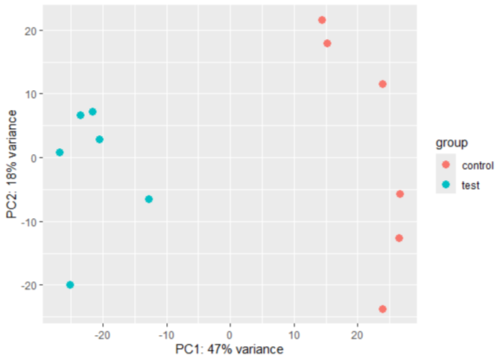

Supplementary Figure S2. Volcano plots of genes in the inoculated platelet concentrate. (A) 0-hour and 1-hour after *S. aureus* inoculation. (B) 0-hour and 3-hour after *S. aureus* inoculation. (C) 0-hour and 6-hour after *S. aureus* inoculation.

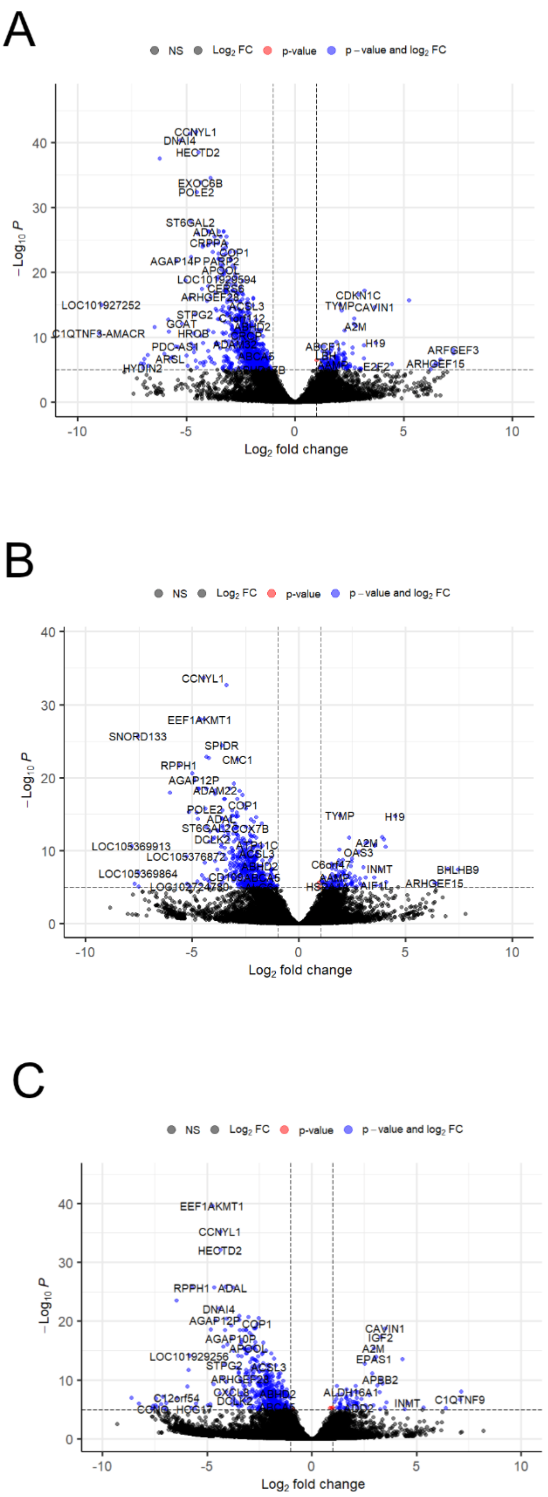

Supplementary Figure S3. (A) Heatmaps of gene expression pattern of inoculated platelet concentrates across all time points. (B) 0-hour and 1-hour after *S. aureus* inoculation. (C) 0-hour and 3-hour after *S. aureus* inoculation. (D) 0-hour and 6-hour after *S. aureus* inoculation.

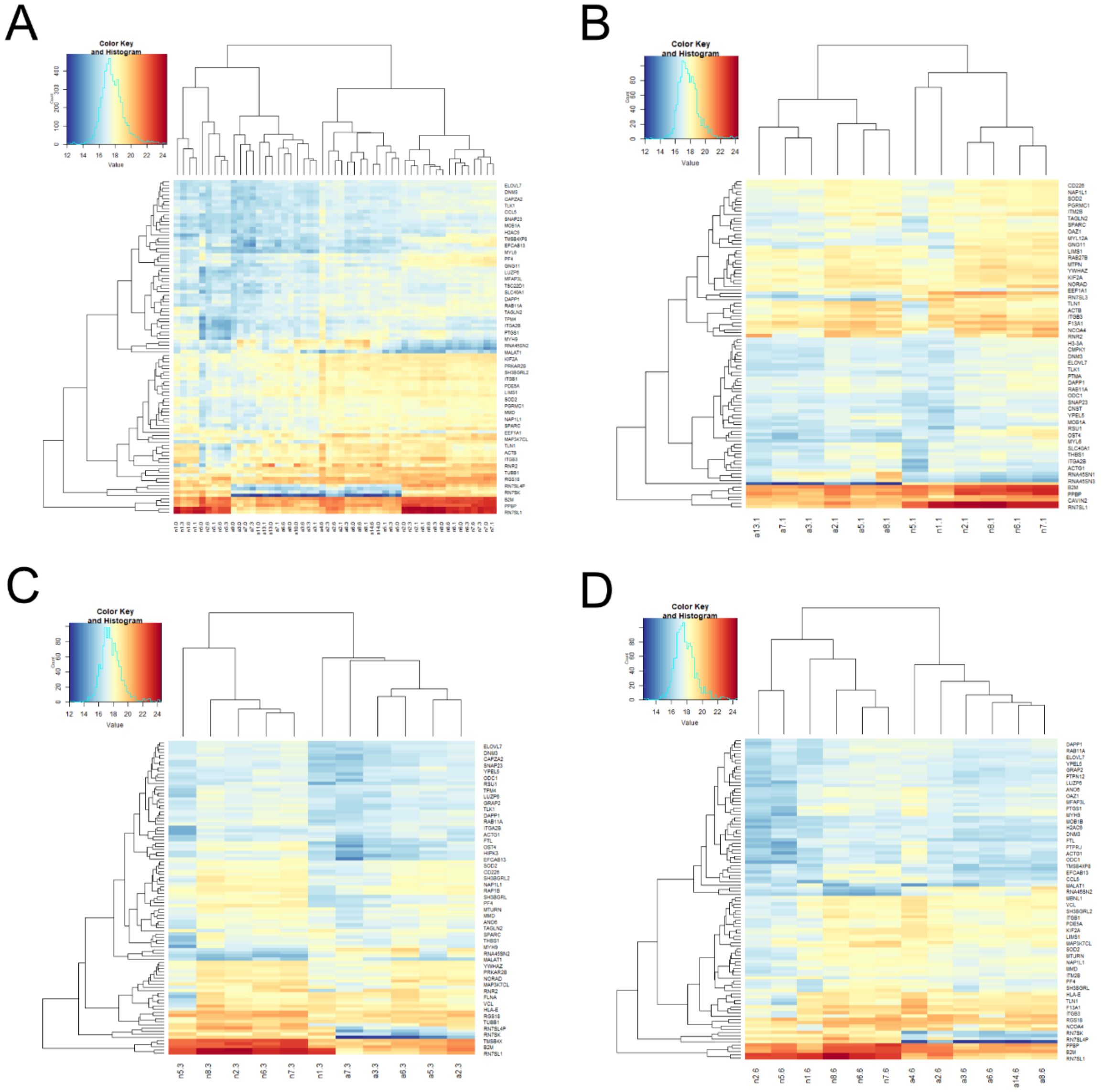

Supplementary Figure S4. Term-gene graph of platelet concetrates inoculated with *S. aureus*. The result shows that two pathways had no overlapping genes.

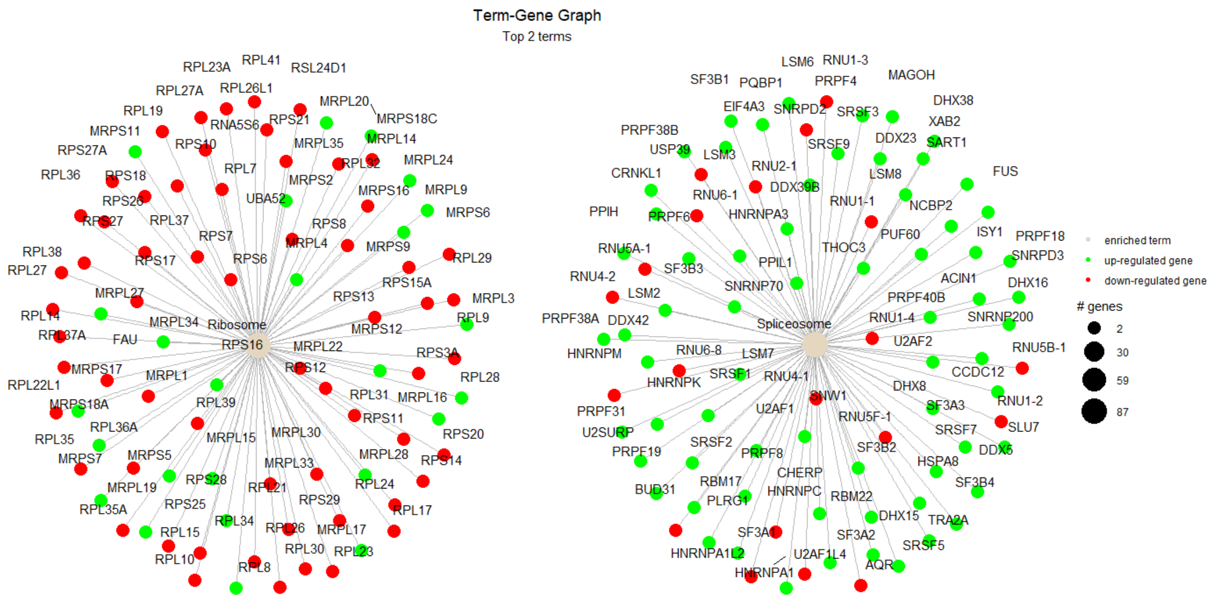

Supplementary Figure S5. Enrichment map of pathway analysis in samples of *S. aureus* inoculation.

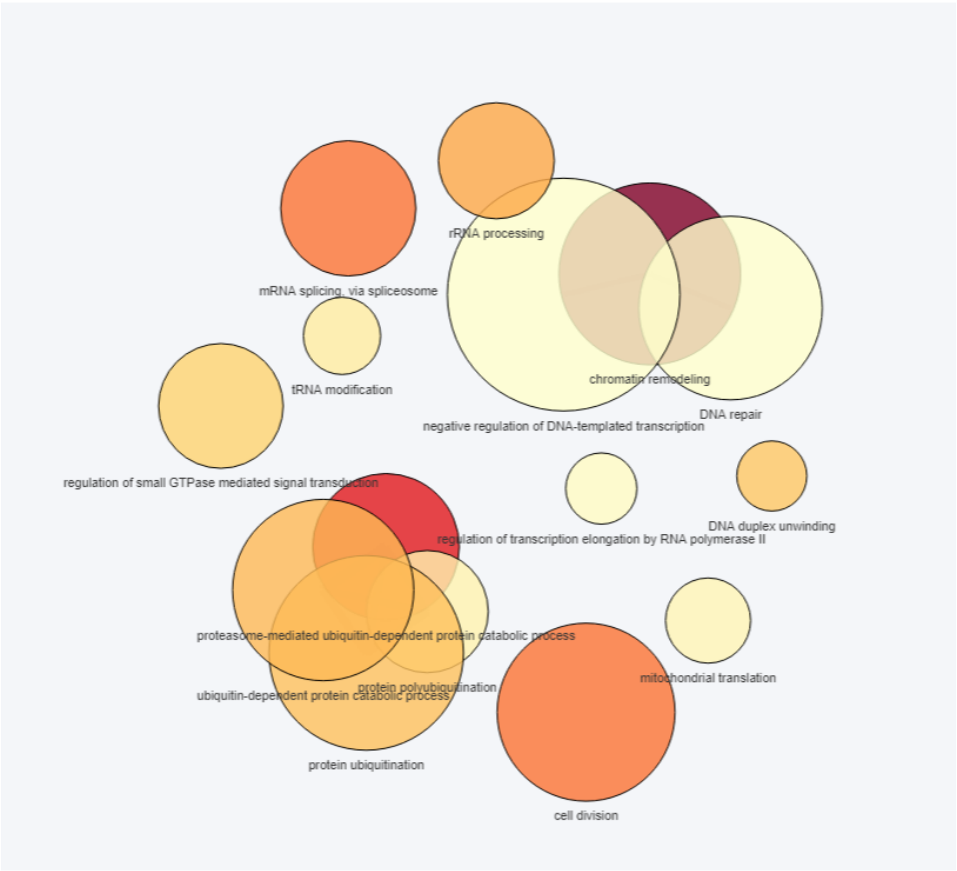

Supplementary Figure S6. Gene set volcano plot of samples inoculated with *S. aureus*.

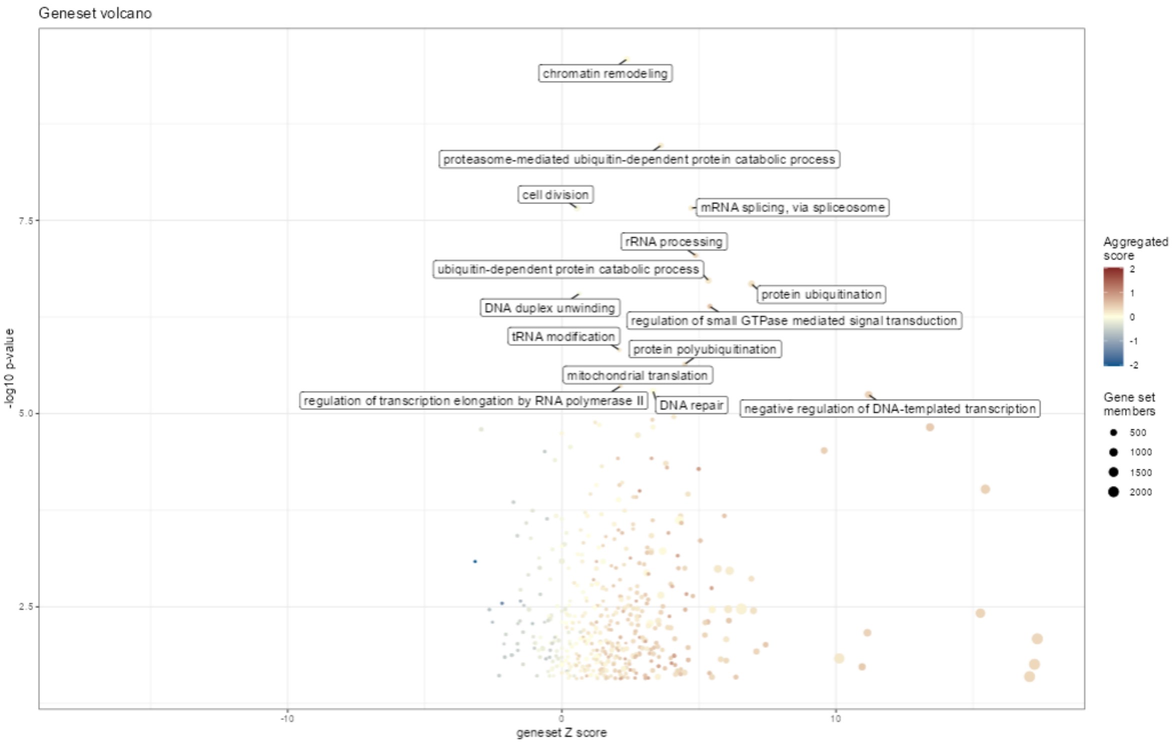

Supplementary Figure S7. PCA plots of gene expression profiles of platelet concentrate after *S. epidermidis* inoculation. (A) 0-hour and 1-hour after *S. epidermidis* inoculation. (B) 0-hour and 3-hour after *S. epidermidis* inoculation. (C) 0-hour and 6-hour after *S. epidermidis* inoculation.

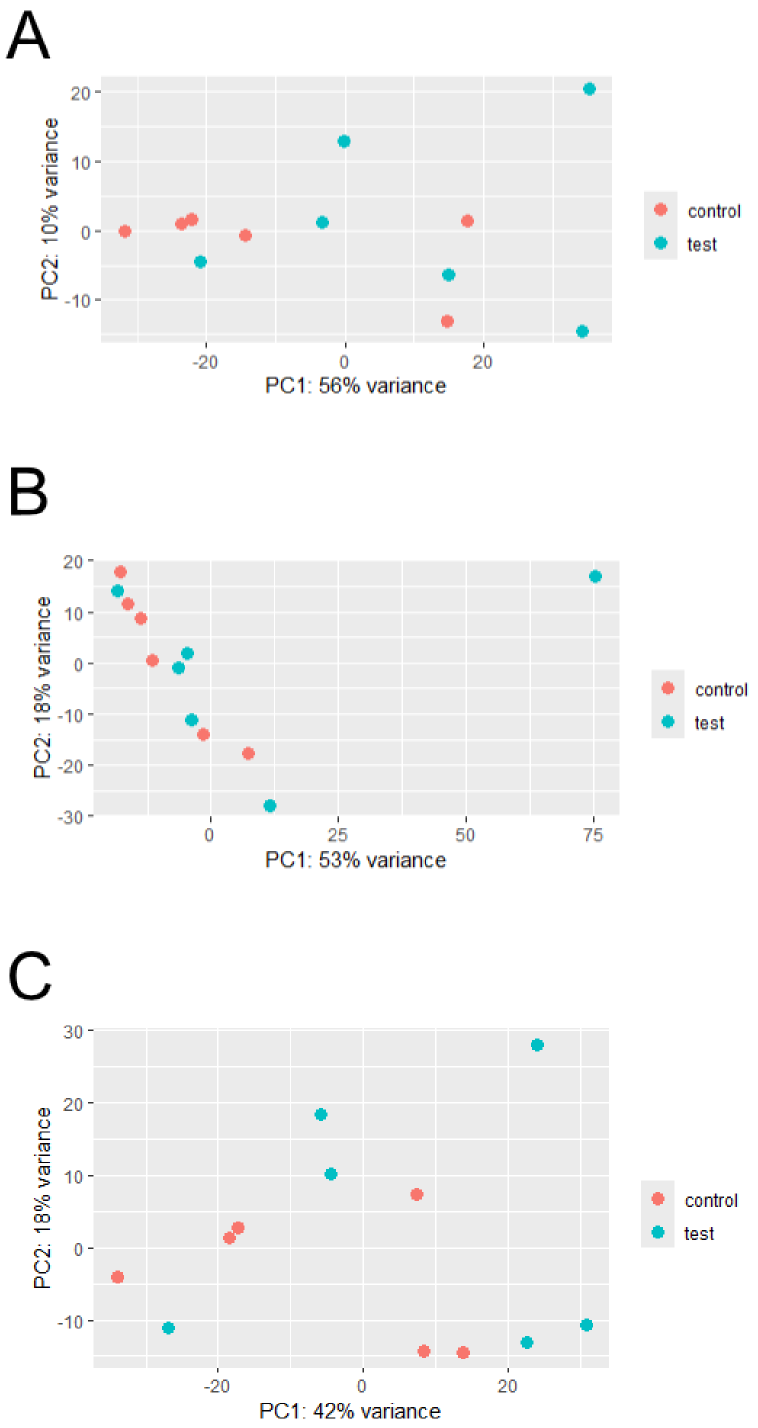

Supplementary Figure S8. Volcano plots of genes in the *S. epidermidis* inoculated platelet concentrate. (A) 0-hour and 1-hour after *S. epidermidis* inoculation. (B) 0-hour and 3-hour after *S. epidermidis* inoculation. (C) 0-hour and 6-hour after *S. epidermidis* inoculation.

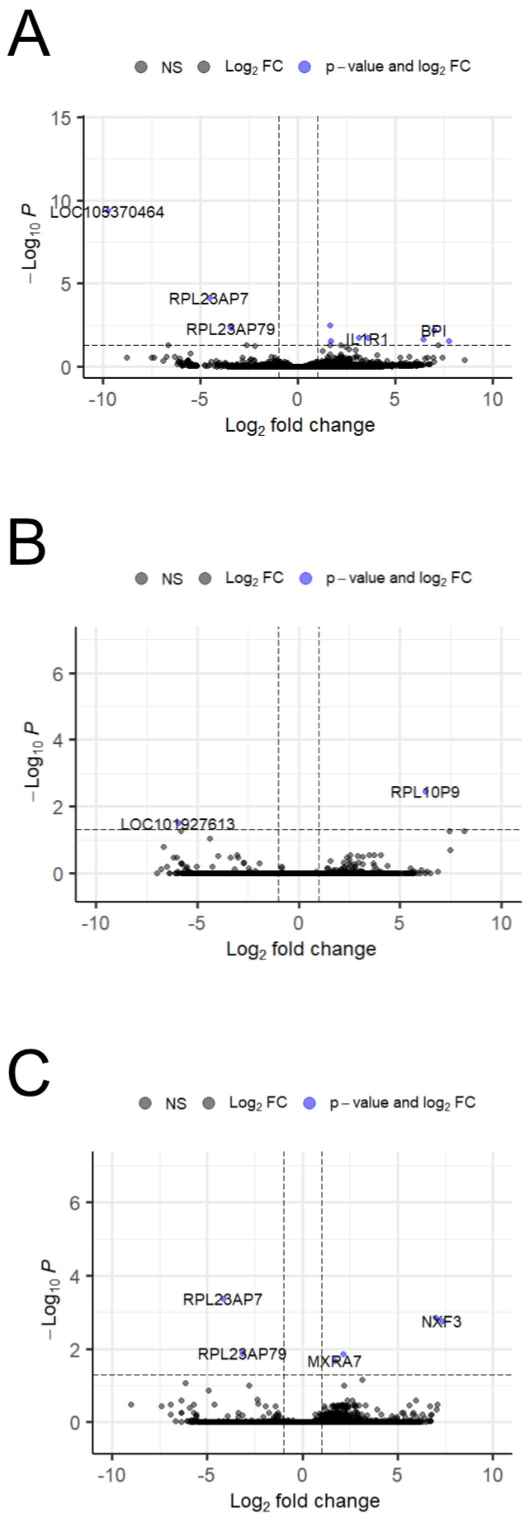

Supplementary Figure S9. Term-gene graph of platelet concetrates inoculated with *S. epidermidis*. The result shows that 4 pathways had several overlapping genes such as *PAK1*, *RAF1*, *GRB2*, and *ARAF*.

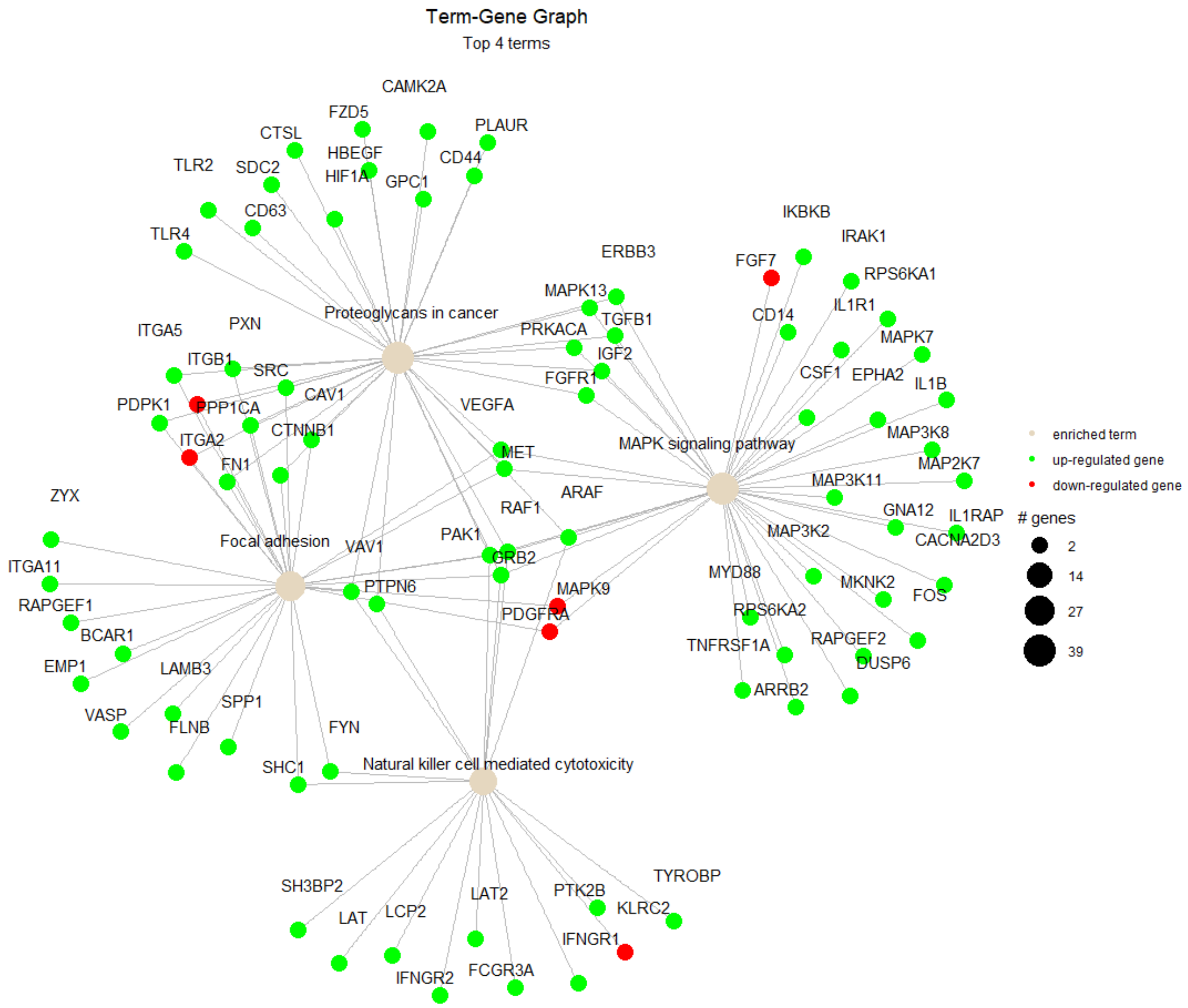

Supplementary Figure S10. Enrichment map of pathway analysis in samples of *S. epidermidis* inoculation.

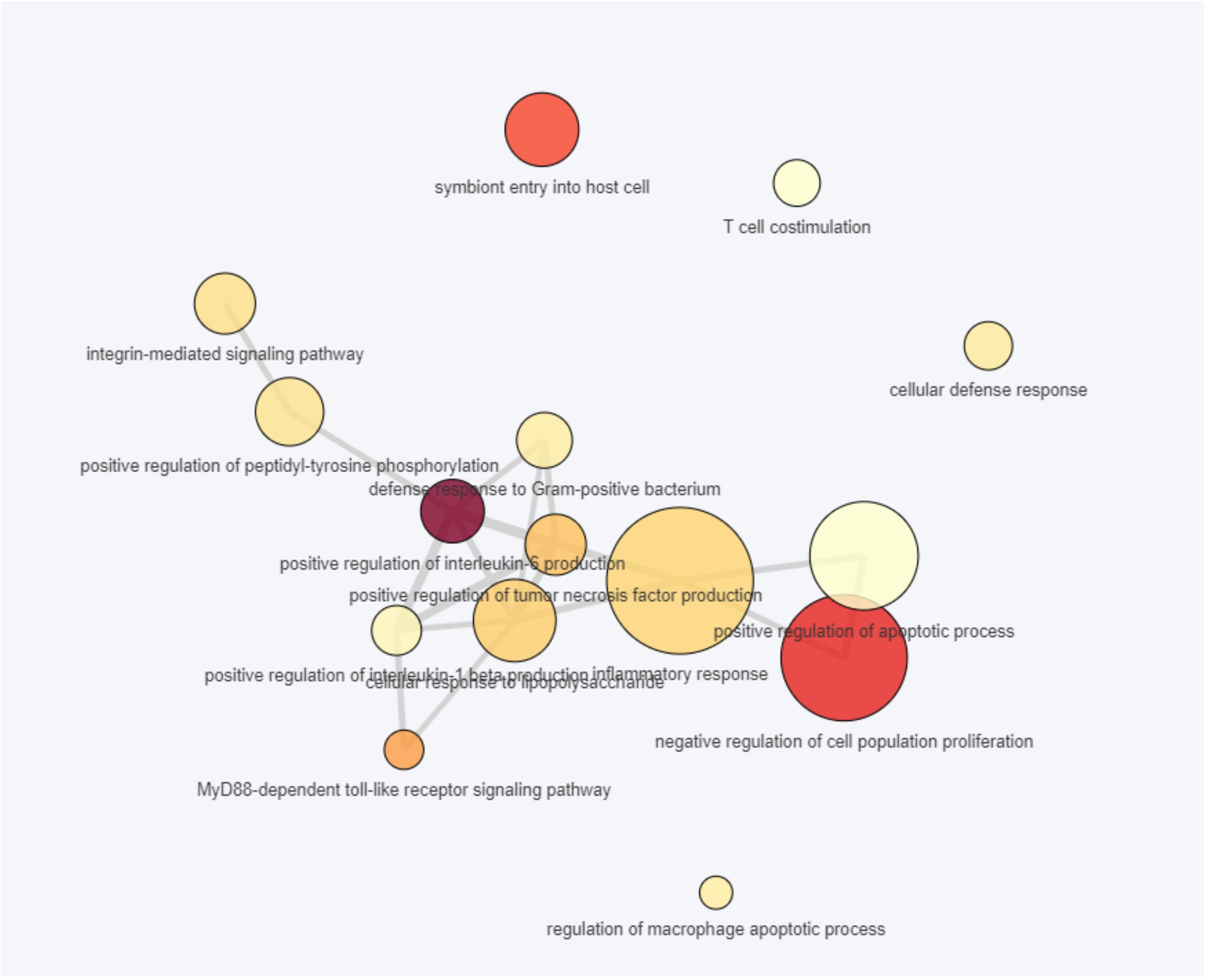

Supplementary Figure S11. The signature heatmap demonstrated enhanced gene expression of the pathway of positive regulation of IL-6 production in *S. epidermidis*-inoculated samples relative to the control group. This differential expression pattern suggests a robust transcriptional response specifically associated with IL-6 production regulation following *S. epidermidis* exposure.

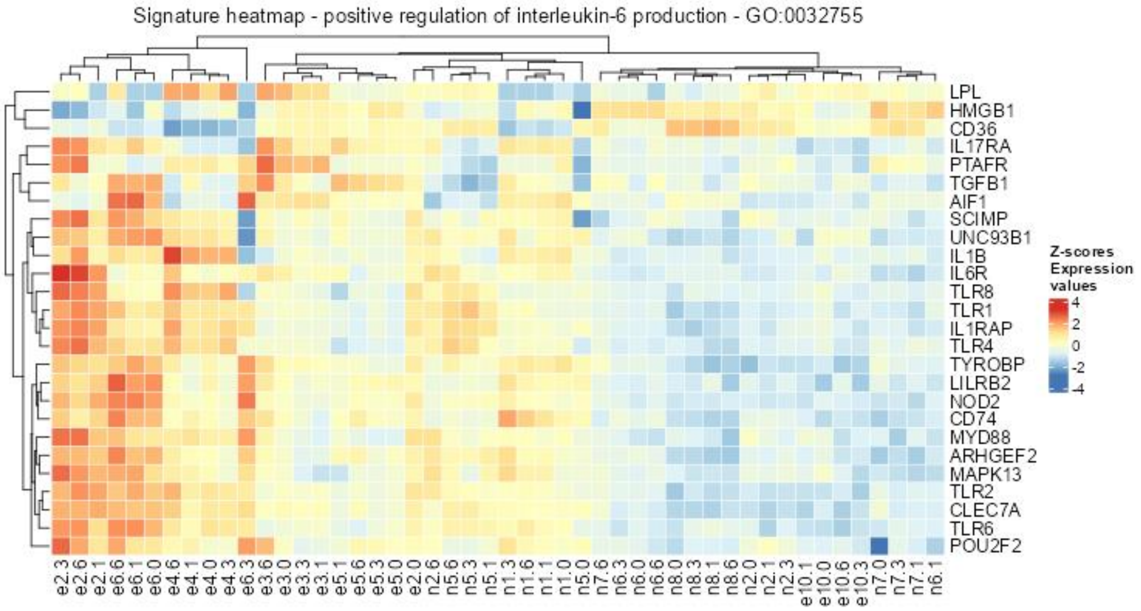

Supplementary Figure S12. Gene set volcano plot of samples inoculated with *S. epidermidis*.

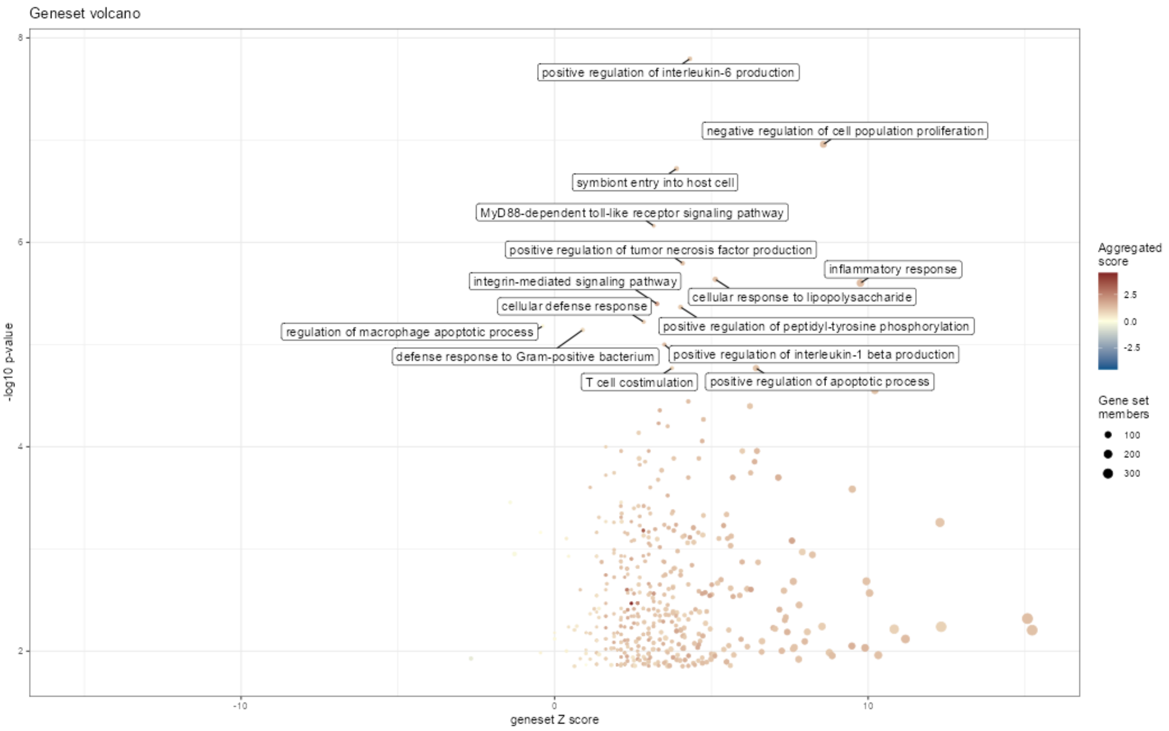

Supplement: Supplementary file 1 [file ijms-26-03009-s001.zip › ijms-3490968-supplementary.pdf]
